# Complete genome sequence of *Bacillus thuringiensis* strain HD521

**DOI:** 10.1186/s40793-015-0058-1

**Published:** 2015-09-04

**Authors:** Qiao Li, Li Z. Xu, Ting Zou, Peng Ai, Gang H. Huang, Ping Li, Ai P. Zheng

**Affiliations:** Rice Research Institute of Sichuan Agricultural University, Chengdu, 611130 China; Key Laboratory of Southwest Crop Gene Resource and Genetic Improvement of Ministry of Education, Sichuan Agricultural University, Ya’an, 625014 China

**Keywords:** *Bacillus thuringiensis*, Gapless chromosome, Parasporal crystal protein, Genomic feature

## Abstract

*Bacillus thuringiensis* is the most widely used biological pesticide in the world. It belongs to the *Bacillus cereus sensu lato* group, which contains six species. Among these six species, *B. thuringiensis*, *B. anthracis*, and *B. cereus* have a low genetic diversity. *B. thuringiensis* strain HD521 shows maroon colony which is different from most of the *B. thuringiensis* strains. Strain HD521 also displays an ability to inhibit plant sheath blight disease pathogen (*Rhizoctonia solani* AG1 IB) growth and can form bipyramidal parasporal crystals consisting of three *cry7* genes. These crystals have an insecticidal activity against *Henosepilachna vigintioctomaculata* larva (Coleoptera). Here we report the complete genome sequence of strain HD521, which has one chromosome and six circular plasmids.

## Introduction

The *B. cereus**sensu lato* group has low genetic diversity when they are measured by multilocus sequence typing and 16S sequencing and some gene contents [1–3]. *B. thuringiensis*, *B. anthracis*, and *B. cereus* are considered one lineage of the *B. cereus* group [4]. The classification of these organisms is based on the differences in their phenotypes and in their pathological effects. The virulence genes are generally located on the plasmids, which obtained them through horizontal gene transfer. These genes give them different phenotypes and pathologies [1]. *B. thuringiensis* is a rod-shaped, Gram-positive, spore-forming bacterium. It produces parasporal protein crystals that show different insecticidal activities against multifarious insect larvae, and some of them exhibit cytocidal activity against cancer cells [5, 6]. *B. thuringiensis* can also produce antibiotics such as Zwittermycin A, which is used to enhance its insecticidal activity and inhibit pathogens fungi, oomycetes, and similar organisms [7–9]. The complete antibiotic biosynthesis gene cluster was first identified in the strain *B. cereus* UW85 [10]. The specific pathology against insects makes *B. thuringiensis* a mainstay of microbial insect control. Although 42 *B. thuringiensis* strains have been sequenced, gapless chromosomes and plasmids have only been obtained from 15 strains’ [11]. Here the complete genome sequence of *B. thuringiensis* strain HD521 is reported and an annotation and description of its genome features is provided. This may provide insight into the genomic diversity among *B. thuringiensis*, *B. anthracis*, and *B. cereus* and the mechanism by which the Zwittermycin A gene cluster was transferred between *B. cereus* and *B. thuringiensis*.

## Organism information

### Classification and features

**Fig. 1:**
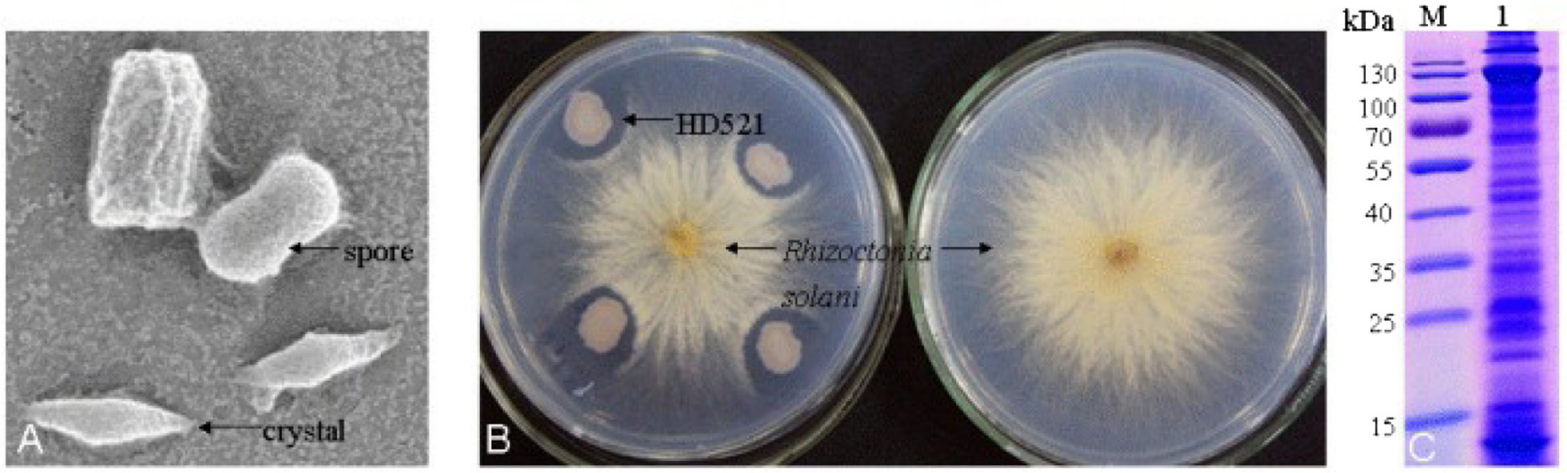
General characteristics of *Bacillus thuringiensis* strain HD521. **a** Scanning electron microscope (SEM) analysis of HD521 spores and parasporal crystals. **b** Antagonism assay of HD521 against Rhizoctonia solani subgroup AG1 IB on PDA medium. **c** SDS-PAGE analysis of spore-crystal suspension of HD521: lane M, molecular mass standard; lane 1, HD521

*B. thuringiensis* strain HD521 was first isolated from soil sample of the United States [12]. It was obtained from *Bacillus* Genetic Stock Center (BGSC). Strain HD521 likes the majority of the *B. thuringiensis* strains, cells are Gram-positive and rod-shaped [5]. It is an aerobic, facultative anaerobic, motile and spore-forming bacterium, with growth temperatures from 10 to 48 °C and optimal growth at 28–35 °C and pH 4.9–8.0 with an optimal pH 7.0 [12–15]. Baumann [16] showed that *B. thuringiensis* strain HD521 utilizes D-glucose, D-ribose, trehalose, pyruvate, glycerol and L-serine and produces extracellular of amylase and gelatinase. Hydrolysis study shows that it has ability to hydrolyze starch, gelatin, glycogen and N-acetyl-glucosamine [17]. It exhibits maroon colonies and produces bipyramidal parasporal crystals during the stationary phase of its growth cycle, which consisted of three *cry7* genes (Fig. 1a). Strain HD521 showed an ability to inhibit *R. solani* AG1 IB growth (Fig. 1b). SDS-PAGE analysis of spores and crystals mixtures showed the strain HD521 expression a major protein band of 130 kDa, which is consistent with the following analysis of its parasporal crystal gene (Fig. 1c). The key features of HD521 are showed in Table 1.

**Table 1:** Classification and general features of *B. thuringiensis* strain HD521 according to the MIGS recommendation [22]

MIGS ID	Property	Term	Evidence code^a^
	Classification	Domain Bacteria	TAS [36]
		Phylum *Firmicutes*	TAS [37]
		Class *Firmibacteria*	TAS [38, 39]
		Order *Bacillales*	TAS [40, 41]
		Family *Bacillaceae*	TAS [40, 42]
		Genus *Bacillus*	TAS [40, 43]
		Species *Bacillus thuringiensis*	TAS [40, 44]
		(Type) strain: ATCC10792	
	Gram stain	Positive	NAS
	Cell shape	Rod	IDA
	Motility	Motile	TAS [14]
	Sporulation	Spore type	IDA
	Temperature range	10 °C to 48 °C	TAS [14]
	Optimum temperature	28 °C–35 °C	TAS [14]
	pH range; Optimum	4.9–8.0; 7.0	TAS [15, 37]
	Carbon source	Organic carbon source	NAS
MIGS-6	Habitat	Soil	TAS [12]
MIGS-6.3	Salinity	Salt-tolerant	TAS [13]
MIGS-22	Oxygen requirement	Aerobic, facultative anaerobic	TAS [16]
MIGS-15	Biotic relationship	free-living	IDA
MIGS-14	Pathogenicity	Insect pathogen	TAS [5]
MIGS-4	Geographic location	United States	TAS [12]
MIGS-5	Sample collection	1981	TAS [12]
MIGS-4.1	Latitude	unreported	TAS [12]
MIGS-4.2	Longitude	unreported	TAS [12]
MIGS-4.4	Altitude	unreported	TAS [12]

^a^Evidence codes - IDA: inferred from direct assay; TAS: traceable author statement (i.e., a direct report exists in the literature); NAS: non-traceable author statement (i.e., not directly observed for the living, isolated sample but rather based on a generally accepted property for the species or on anecdotal evidence). These evidence codes are from the Gene Ontology project [45]. If the evidence is IDA, then the property was directly observed for a live isolate by one of the authors or an expert mentioned in the acknowledgements

**Fig. 2:**
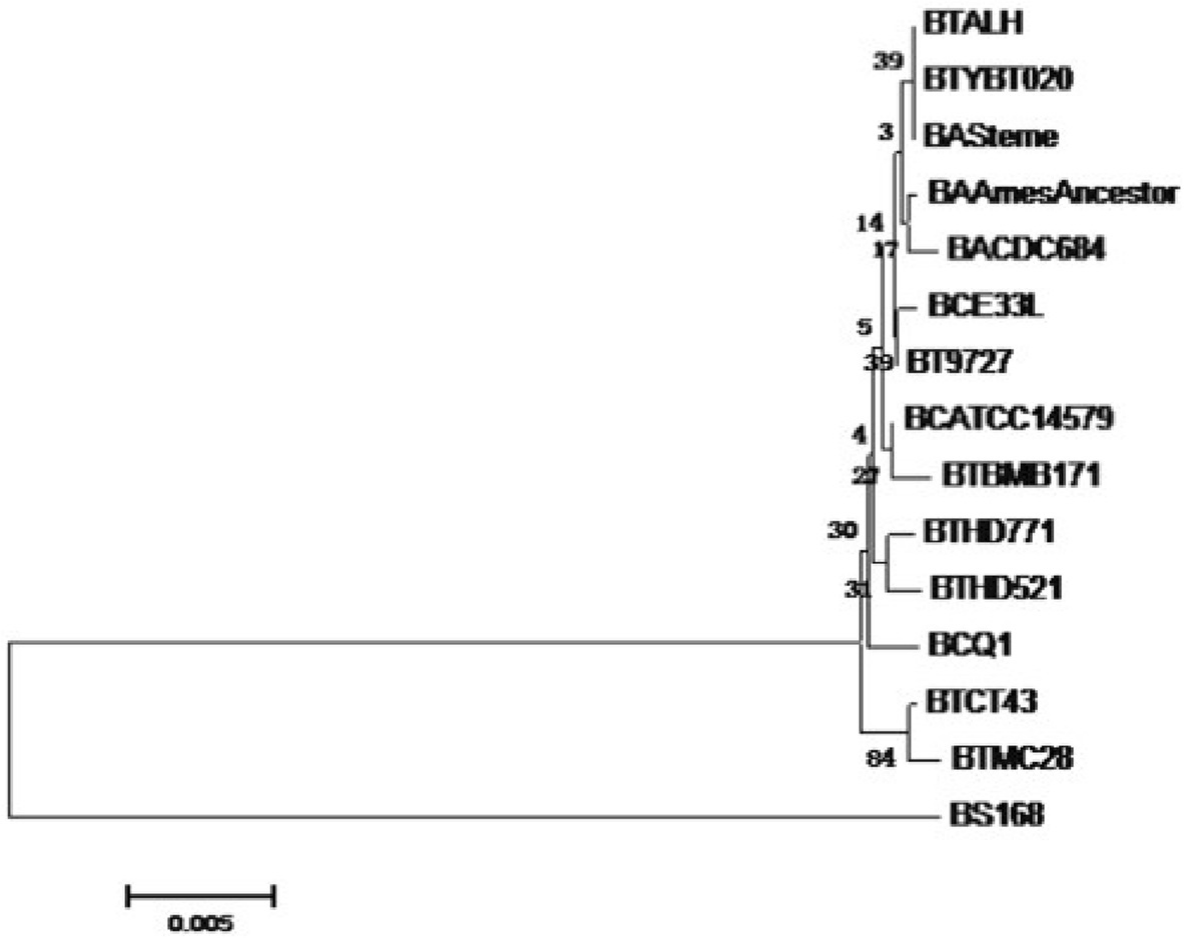
Neighbor-joining phylogenetic tree highlighting the position of *B. thuringiensis* HD521 relative to *B. thuringiensis*, *B. anthracis*, and *B. cereus*. The strains and their 16 s rRNA corresponding to the GenBank accession numbers given below: A) BTALH (*B. thuringiensis str. AL Hakam*) (CP000485.1): 9309–10762; B) BTYBT020 (*B. thuringiensis serovar finitimus* YBT-020) (CP002508.1): 9307–10866; C) BASterne (*B. anthracis str*. Sterne ) (AE017225.1): 9336–10845; D) BAAmesAncestor (*B. anthracis str*. ‘Ames Ancestor’) (AE017334.2): 29129–30635; E) BACDC684 (*B. anthracis str*. CDC684) (CP001215.1): 9207–10715; F) BCE33L (*B. cereus* E33L) (CP_000001.1): 9337–10846; G) BT9727 (*B. thuringiensis serovar konkukian str*. 97–27) (AE017355.1): 9337–10846; H) BCATCC14579 (*B. cereus* ATCC14579) (AE016877.1): 9186–10741; I) BTBMB171 (*B. thuringiensis* BMB171) (CP001903.1): 9197–10736; J) BTHD771 (*B. thuringiensis* HD-771) (CP003752.1): 4786891–4788450; K) BTHD521 (*B. thuringiensis subsp. indiana* HD521) (CP010106): 9198–10737; L) BCQ1 (*B. cereus* Q1) (CP000227.1): 9338–10843; M) BTCT43 (*B. thuringiensis serovar chinensis* CT-43) (CP001907.1):9201–10740; N) BTMC28 (*B. thuringiensis* MC28) (CP003687.1):1369440–1370979; O) BS168 (*B. sublitis subsp. str.* 168) (CM000487.1): 9808–11362

Fourteen strains and HD521 were chosen for phylogenetic analysis. They showed a sequence similarity of more than 97 % based on blast analysis [18]. A 16 s rRNA sequence from *B. subtilis* 168 was selected as outgroup. The maximum likelihood method was used to construct the phylogenetic tree and the phylogenetic relationship of these 15 strains is shown in Fig. 2. Phylogenetic tree shows that strain HD521 has a close genetic relationship to strain HD771. The bootstrap value of this Phylogenetic tree is very low because of the 16S rRNA nucleotide sequence divergence of the chosen strains is low which is accordance to the previous studies. Ash showed that 16S rRNA nucleotide sequences among *B. cereus*, *B. thuringiensis* and *B. anthracis* were high similar and exhibit more than 99 % similarity [19], and they are considered as a single species [4, 20, 21].

## Genome sequencing information

### Genome project history

Studies of cytological and biological activity have provided three reasons to select it for sequencing of its whole genome: 1) Strain HD521 produces maroon colonies, unlike most of the *B. thuringiensis* strains. It can also form bipyramidal parasporal crystals and shows insecticidal activity against the larva of *Henosepilachna vigintioctomaculata* (Coleoptera). 2) Strain HD521 shows an ability to inhibit the growth of the pathogenic fungus *R. solani* AG1 IB and to provide information regarding the mechanism of antibiotic gene cluster transfer between *B. thuringiensis* and *B. cereus*. 3) Until now, the genomes of only 15 strains of *B. thuringiensis* have been completed. No *B. thuringiensis* serovar *Indiana* strain has been fully sequenced. The complete sequence of HD521 may contribute to the evolution and comparative genomics of the *B. thuringiensis* and *Bacillus cereus**sensu lato* group. The complete gapless chromosome sequence and sequences of 6 plasmids sequence have been deposited in GenBank under the accession numbers of CP010106, CP010107, CP010108, CP010109, CP010110, CP010111 and CP010112. A summary of the genome sequencing project information has been deposited in the Joint Genome Institute with MIGS version 2.0 under the ID of Gp0111431 [22]. The summary of the detail information is shown in Table 2.

**Table 2:** Project information

MIGS ID	Property	Term
MIGS 31	Finishing quality	Complete
MIGS-28	Libraries used	Three libraries, two Illumina paire-end libraries (180 bp inserted size; 500 bp inserted size); one Fosmid library
MIGS 29	Sequencing platforms	Illumina HiSeq 2000, Sanger 3730
MIGS 31.2	Fold coverage	377×
MIGS 30	Assemblers	Velvet 1.2.10 version
MIGS 32	Gene calling method	GeneMarkS
	Locus Tag	NF53
	Genbank ID	CP010106
	GenBank Date of Release	20-July-2015
	GOLD ID	Gi0079964
	BIOPROJECT	PRJNA263441
MIGS 13	Source Material Identifier	*Bacillus* Genetic Stock Center
	Project relevance	Chinese Major Project

### Growth conditions and DNA preparation

One colony was picked from LB plate medium and growth in 50 ml LB fluid medium overnight at 180 rpm, 30 °C. Cells were collected by centrifugation and washed with 20 ml cold TES buffer twice (30 mM Tris base, 5 mM EDTA, 50 mM NaCl; pH = 8.0) and then resuspended in 7.2 ml TES buffer with 20 % sucrose, lysozyme (20 mg/ml) and RNase A (1 μl/ml) and then incubated at 37 °C for 3–4 h. 7.2 ml TES with 8 % sodium dodecyl sulfate (SDS) was added in the spheroplast suspension and incubated at 68 °C for 10 min. Then 3.6 ml of 3 M sodium acetate (PH = 4.8) was added and the total suspension was incubate at −20 °C for 30 min. The suspension was centrifuged at 18,000 × g for 20 min at 4 °C. Supernatant was transferred into a new centrifuge tube and then centrifuged at 18,000 × g for 20 min at 4 °C again. Two volumes of cold absolute ethanol were added to the supernatant and incubated at −20 °C for about 12 h. DNA was pelleted at 18,000 × g for 20 min at 4 °C and pellet was dissolved in 300 μl sterile double distilled water and stored at −20 °C for further use. All of the operations were according to the previous report [23].

### Genome sequencing and assembly

The genome of HD521 was sequenced at Beijing Genomics Institute (BGI; Shenzhen, China) using Illumina HiSeq 2000 platform with two paired-End Modules. Sequencing of 500 bp paired-end modules gathered 3.88 M reads, about 58 fold coverage (0.36 Gb); sequencing of 180 bp paired-end modules gather 14.76 M reads, about 319 fold coverage (2 Gb). These data were *de novo* assembled with Velvet, version 1.2.10 [24]. The assembly finally resulted in 77 scaffolds. Possible circular scaffolds were verified by PCR. The precedence relationships among the remainder scaffolds were predicted by using Nucleotide BLAST with the beginning and the end sequences of each scaffold. A Fosmid library was constructed and used to confirm some long-distance connected relations between corresponding scaffolds. Gap closing was using primer walking. Finally, 186 correct sub-clones were used to close gaps among the possible connected scaffolds. Anteroposterior sequences of the gaps were used for primer design directly for inner gap closing, 13 sub-clones were used for inner gap closing. Finally one gapless chromosome and six plasmids were obtained.

### Genome annotation

Open reading frames were called used GeneMarkS with the model parameter trained on the complete sequence [25]. The predicted ORFs were translated and searched in the National Center for Biotechnology Information non-redundant database and then annotated to PFAM, GO, KEGG, Swiss-Prot, COG, and TrEMBL databases. The NR, KEGG, Swiss-Prot, and TrEMBL databases were annotated using Blast and e-values of 1e-50, and each protein was selected using the best hit. PFAM was annotated using InterProScan, and GO was annotated using Blast2GO with the NR database annotation. The rRNA, tRNA, and sRNA were predicted using rRNAmmer, tRNAscan, and Rfam, respectively [26–28]. Genes with signal peptides and transmembrane helices were predicted using SignalP, version 3.0 and TMHMM, version 2.0 [29, 30]. CRISPER repeats were predicted by using CRISPRfinder [31, 32].

## Genome properties

**Fig. 3:**
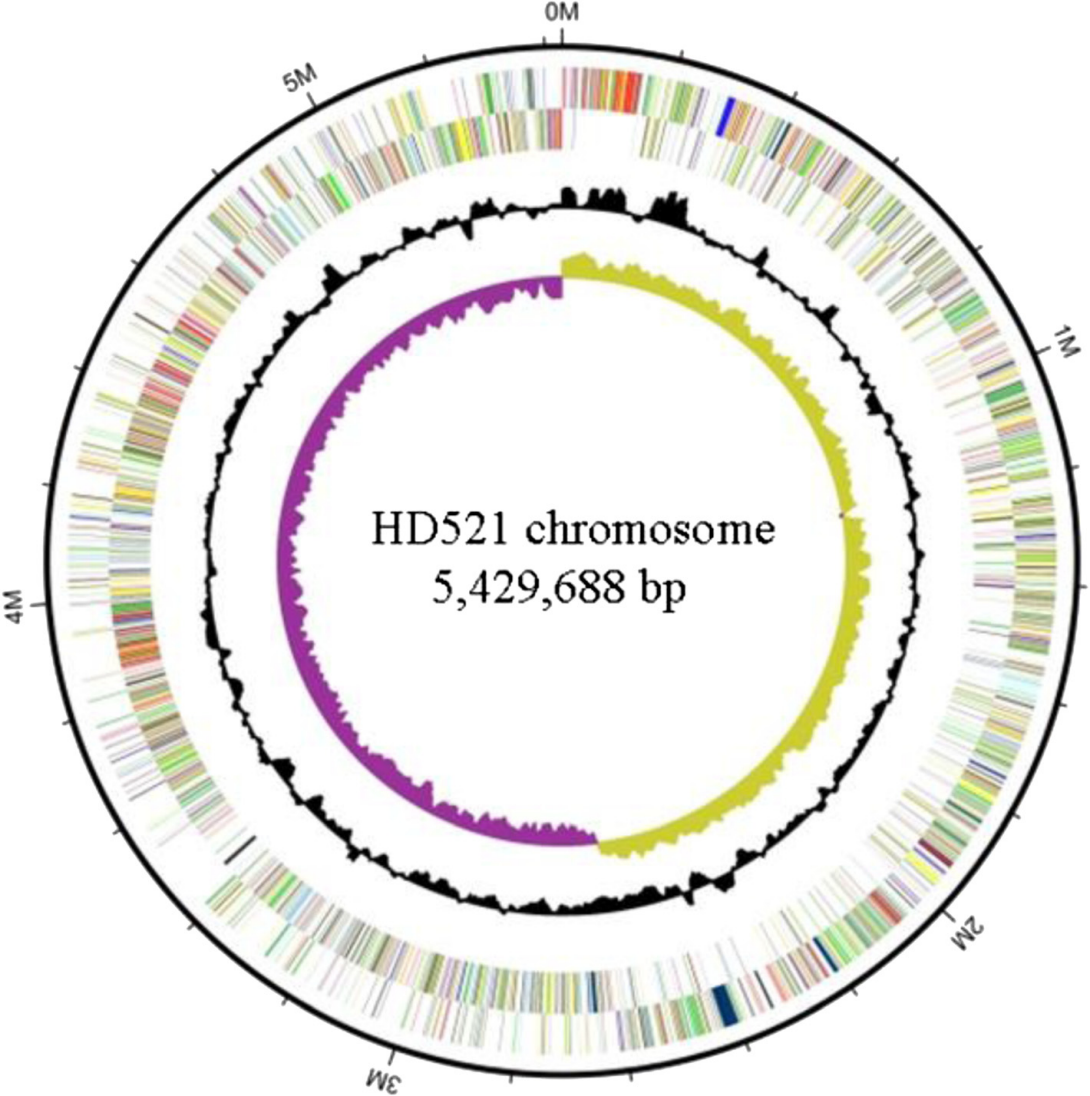
Circular representation of chromosome of HD521 performing relevant genome features. From outside to center; Genes on forward strand (colored genes annotated into COG categories), Genes on reverse strand (colored genes annotated into COG categories), G + C content (black), and G + C skew

**Fig. 4:**
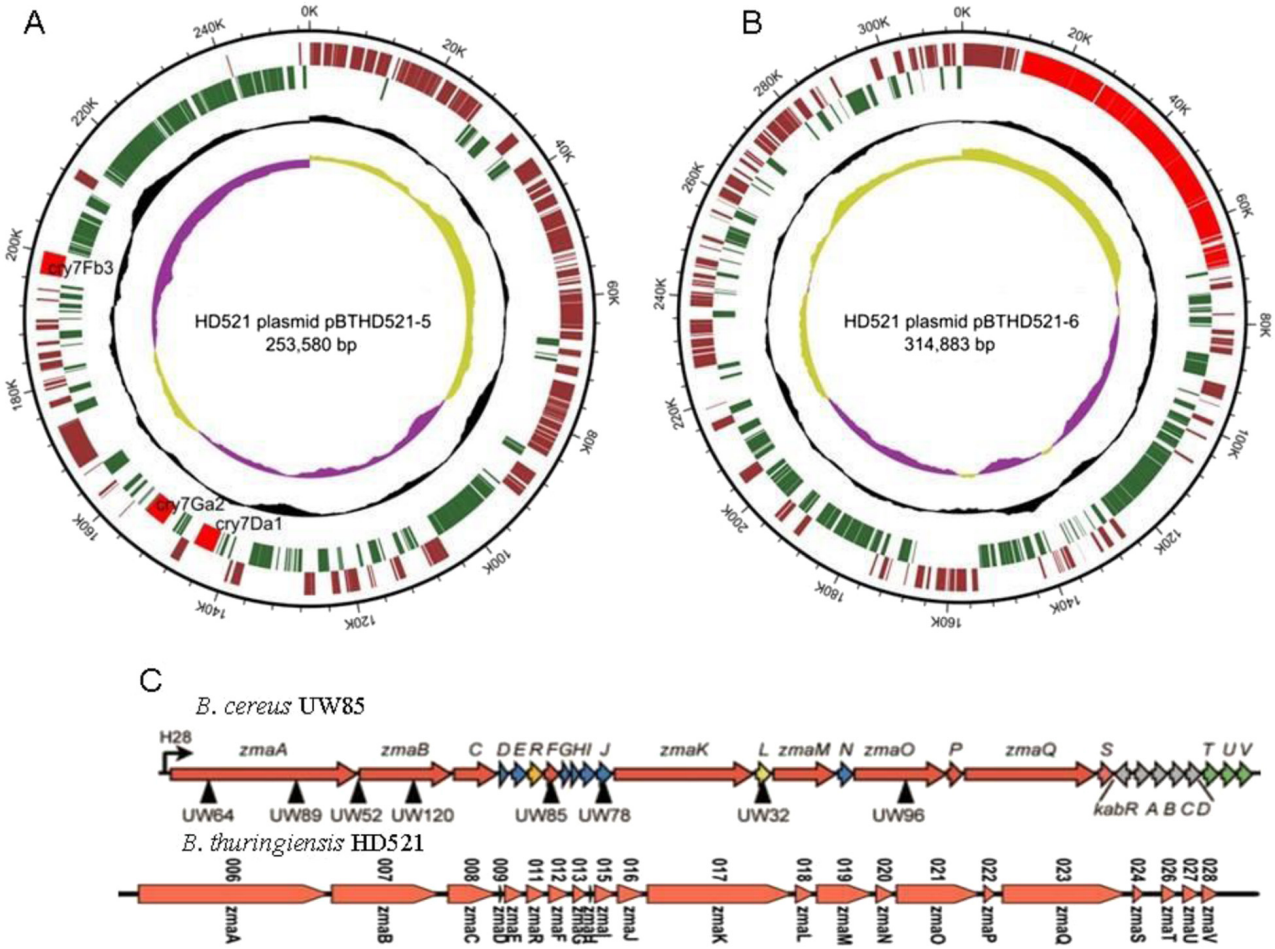
Circular representation of plasmids pBTHD521-5 and pBTHD521-6. **a** and **b** Circular representation of plasmid pBTHD521-5 and pBTHD521-6 displaying relevant genome features. From outside to center: Genes on forward strand (dark red by COG categories), genes on reverse strand (green by COG categories), G + C content (black) and G + C skew. Red regions of pBTHD521-5 represent three *cry7* genes: *cry7Fb3* (KF672184), *cry7Ga2*, and *cry7Da1*; red region of pBTHD521-6 represents the Zwittermycin A gene cluster. **c** comparison of Zwittermycin A gene cluster between *B. cereus* UW85 and *B. thuringiensis* HD521 [10]. The figure of *B. cereus* UW85 Zwittermycin A gene cluster is cited from reference paper 10

The genome of HD521 consisted of 7 replicons: a circular chromosome with a length of 5,429,688 bp (Fig. 3, Table 3). The G + C content of the circular chromosomes was 35.28 %. It included a predicted 5,538 genes 138 are RNA genes. Of these 5,400 genes, with a collective length of 4,544,493 bp, were protein-encoding genes. Table 4 displays the six circular plasmids pBTHD521-1, pBTHD521-2, pBTHD521-3, pBTHD521-4, pBTHD521-5 (Fig. 4a), and pBTHD521-6 (Fig. 4b). The G + C contents of the six plasmids ranged from 29.45 to 35.91 % and contained a total of 772 predicted genes. The plasmid pBTHD521-5 contained three *cry7* genes, which can form bipyramidal parasporal crystals (data not shown). Among all the predicted genes, 3,323 were placed in 25 general COG function gene catalogs. The distribution of the predicted genes, which are annotated with COG functional categories, is presented in Table 5.

**Table 3:** Genome statistics

Attribute	Value	% of Total
Genome size (bp)	5,429,688	100.00
DNA coding (bp)	4,544,493	83.69
DNA G + C (bp)	1,915,594	35.28
DNA scaffolds	77	
Total genes	5,538	100.00
Protein coding genes	4,544,493	83.69
RNA genes	138	2.49
Pseudo genes	0	0.00
Genes in internal clusters	0	0.00
Genes with function prediction	4,308	77.79
Genes assigned to COGs	3,323	60.00
Genes with Pfam domains	4,462	80.57
Genes with signal peptides	941	16.69
Genes with transmembrane helices	1,576	28.46
CRISPR repeats	0	0.00

**Table 4:** Summary of genome: one chromosome and 6 plasmids

Label	Size (Mb)	Topology	INSDC identifier	RefSeq ID
Chromosome	5.40	circular	CP010106	CP004069.1
Plasmid 1	0.007	circular	CP010107	AB083655.3
Plasmid 2	0.050	circular	CP010108	CP004871.1
Plasmid 3	0.072	circular	CP010109	CP002178.1
Plasmid 4	0.072	circular	CP010110	CP004071.1
Plasmid 5	0.25	circular	CP010111	CP007615.1
Plasmid 6	0.31	circular	CP010112	CP007616.1

**Table 5:** Number of genes associated with the 25 general COG functional categories

Code	Value	% of total^a^	Description
J	221	4.3	Translation
A	0	0.00	RNA processing and modification
K	473	9.27	Transcription
L	222	4.35	Replication, recombination, and repair
B	2	0.04	Chromatin structure and dynamics
D	43	0.84	Cell cycle control, mitosis, and meiosis
Y	0	0.00	Nuclear structure
V	122	2.39	Defense mechanisms
T	368	7.21	Signal transduction mechanisms
M	262	5.13	Cell wall/membrane biogenesis
N	62	1.21	Cell motility
Z	0	0.00	Cytoskeleton
W	1	0.02	Extracellular structures
U	59	1.16	Intracellular trafficking and secretion
O	117	2.29	Posttranslational modification, protein turnover, chaperones
C	228	4.47	Energy production and conversion
G	295	5.78	Carbohydrate transport and metabolism
E	491	9.6	Amino acid transport and metabolism
F	125	2.45	Nucleotide transport and metabolism
H	180	3.53	Coenzyme transport and metabolism
I	134	2.60	Lipid transport and metabolism
P	328	6.43	Inorganic ion transport and metabolism
Q	126	2.47	Secondary metabolites biosynthesis, transport and catabolism
R	798	15.63	General function prediction only
S	448	8.78	Function unknown
-	1708	30.84	Not in COGs

^a^ The total is based on the total number of protein coding genes in the annotated genome

## Conclusions

*B. thuringiensis*, *B. cereus*, and *B. anthracis* have a close relationship as indicated by genomes and they are considered the same species [4]. Through the 15 complete *B. thuringiensis* genomes, 15 complete *B. cereus* genomes and 15 *B. anthracis* genomes, the *B. thuringiensis*, *B. cereus*, and *B. anthracis* genomes ranged in size from 5.3 Mbp to 6.7 Mbp (G + C content ranged from 34.91 to 35.41 %), 5.2 Mbp to 5.8 Mbp (G + C content 35.05 to 35.54 %), and 5.2 Mbp to 5.5 Mbp (G + C content 35.26 to 35.4 %), respectively [11, 33, 34]. *B. thuringiensis* has a larger genome than *B. cereus* and *B. anthracis*. The genome of *B. thuringiensis* strain HD521 consisted of a circular chromosome 5.4 Mbp in length and six plasmids ranging from 7,042 bp to 314,882 bp in size for a total genome 6.2 Mbp in length. There were 5,400 protein-encoding genes, 3,323 of which could be assigned to COG functional categories. Among these categories, 9.62 % of the genes were annotated to amino acid transport and metabolism, 9.27 % to transcription, 7.21 % to signal transduction mechanisms, 6.43 % to inorganic ion transport and metabolism, 5.78 % to inorganic ion transport and metabolism carbohydrate transport and metabolism, and 5.13 % to cell wall and membrane biogenesis. *B. thuringiensis* is an insect pathogen, *B. cereus* is an opportunistic human pathogen, and *B. anthracis* is a mammalian pathogen. The differences in their pathogenicity were caused by virulent components located in the plasmids. These were acquired by horizontal gene transfer [1, 4]. The pathogenicity of *B. anthracis* is caused by two plasmids, pXO1 and pXO2 [35]. *B. thuringiensis* and *B. cereus* are more similar to each other, the determinate difference is the insecticidal toxin genes, which are usually located on plasmids [4]. *B. thuringiensis* HD521 contains six plasmids, named pBTHD521-1 through pBTHD521-6. These plasmids each contain 11, 70, 89, 103, 243, and 256 protein-coding genes. The G + C content of these six plasmids ranged from 29.45 to 35.91 %. G + C contents of plasmid pBTHD521-1 and pBTHD521-4 were 29.45 and 29.79 %, which were markedly lower than the general G + C content (34.91 to 35.41 %) of *B. thuringiensis*. It is postulated that pBTHD521-1 and pBTHD521-4 were obtained by *B. thuringiensis* HD521 through horizontal gene transfer.

*B. thuringiensis* strain HD521 forms bipyramidal parasporal crystals and has an insecticidal activity against *Henosepilachna vigintioctomaculata* larva (Coleoptera). SDS-PAGE analysis of spore-crystal suspension showed HD521 express one major protein band of 130 kDa which is encoded by three *cry7* genes located on plasmid pBTHD521-5. One *cry7-like* gene showed 99 % identity to cry7Fb genes. It was named *cry7Fb3* by Delta-Endotoxin Nomenclature Committee. The other two cry7-like genes have a 100 % homology to *cry7Ga2* and *cry7Da1*. *B. thuringiensis* strain HD521 also has an ability to inhibit the growth of plant sheath blight disease pathogen (*R. solani* AG1 IB). A Zwittermycin A gene cluster was found on plasmid pBTHD521-6. There were more than 56 kbp sequences matched to Zwittermycin *B. cereus* UW85 was found to produce a specific gene cluster sequence (FJ430564.1, 65 kbp) and this sequence included its main gene components. This indicated that strain HD521 has utility as a biocontrol agent not only against insect larva but also against plant disease. The complete genome sequence of HD521 may provide another model to study pathogenicity against pests, plant disease, and phylogenesis among *Bacillus cereus**sensu lato* group.
